# At What Moment Does the Rational Soul Enter the Body?

**Published:** 1886-07-20

**Authors:** John J. Gaynor

**Affiliations:** St. John, N. B.


### AT WHAT MOMENT DOES THE RATIONAL SOUL ENTER THE BODY?

BY JOHN J. GAYNOR, M. D., ST. JOHN, N. B.

That any physician can doubt the correct answer to the foregoing question, cannot be food for wonder, because it is seldom that an American medical man possesses sound religious views on medico-moral questions.

1. After thus showing cause for that mental squint which perverts the moral vision of a large class of medical men, the question still remains: At what moment does the rational soul enter the body? I reply, unhesitatingly, *At the moment of conception!* The presence of a human soul in the impregnated human ovule is as essential to fecundation, to life, growth and development, as the union of the male and female elements. "The body, without the spirit, is dead," is an inspired truism without a qualifying restriction, and, consequently, holds good in all cases, no matter whether that body be *intra*-uterine or extra-uterine. Therefore, a soul is essential to life; where life is, a soul is.

2. A part exists by reason of a whole, and a whole contains each one of its parts. A soul is a part of man, a soul exists on account of man, and a man contains a soul; therefore, when Nature wishes to beget a man, and the time arrives to beget that man, a soul must be created that a man be begotten.

Corollary: *A wilfully-provoked abortion is—wilful murder.*

I have already refuted the sophisms that, in the materialistic sense, "the ovum receives its *animal* life by becoming impregnated by the male," and that "it cannot possibly be claimed that the soul takes possession" at that time. Provided, even, I had not the admission that the impregnated ovum is endowed with *animal* life would in itself be a sufficient refutation of both statements. If endowed with "animal life," the impregnated ovum is a live animal. If it is a live animal, it is according to its kind—rational; therefore, it possesses a rational soul.

It is said that the embryo cannot possess a soul, because, forsooth, it gives no evidence of being endowed with "mental faculties, reasoning powers," etc. I respond: "*Nihil est in intellectu quod non fuerit in sensu.*" Fresh from the Creator, without a single experience, where are the subjects for reason? Without a single organ of special sense, whence comes the food for thought? The soul of the embryo is a soul *in embryo.* While being endowed with all its faculties, each faculty is in the germ stage, and, as might be expected, developed parallel with its corresponding organ of expression, with the needs of existence, with the advancement of physical perfection. Thus, at the moment of conception, the vegetative (not "animal") faculties of the soul are the only ones in action. Then, as physical development advances, the sentient, the motor, the rational faculties are called into play.

I have already refuted the false construction put on Gen. ii, 7, but let me add a few words more. If Adam was breathing, he was alive, was already endowed with life. If alive, endowed with life, God would not have had to breathe life into him; therefore, Adam *did not* breathe before he received "the breath of life." Therefore, *the babe cannot breathe without a soul*, or, in other words, the babe receives a soul before it breathes. Ergo: *Craniotomy is homicide.*

I accept the following conclusions as inevitable: (1) The human soul is the vital principle of embryonic life in man. (2) The soul enters the human embryo at the moment of conception. (3) The human embryo, being possessed of a human soul, is an individual human being, and, as such, cannot be deprived of its right to live. (4) The wilful destruction of embryonic life is wilful murder. (5) Craniotomy is homicide.—*Physicians' and Surgeons' Investigator.*

